# Hospital Spiritual Care Can Complement Graduate Medical Trainee Well-Being

**DOI:** 10.1155/2019/8749351

**Published:** 2019-12-10

**Authors:** Robert E. Shapiro, Manuel C. Vallejo, Sarah H. Sofka, Rebecca M. Elmo, Allison H. Anderson, Norman D. Ferrari

**Affiliations:** ^1^West Virginia University School of Medicine, Department of Obstetrics & Gynecology, P.O. Box 9186, Robert C. Byrd Health Sciences Center, Morgantown, WV 26506, USA; ^2^West Virginia University School of Medicine, Departments of Medical Education and Anesthesiology, P.O. Box 9001A, Robert C. Byrd Health Sciences Center, Morgantown, WV 26506, USA; ^3^West Virginia University School of Medicine, Department of Medicine, 4th Floor HSCN Rm 4086, Morgantown, WV 26506, USA; ^4^West Virginia University School of Medicine, Department of Medical Education, P.O. Box 9001A, Robert C. Byrd Health Sciences Center, Morgantown, WV 26506, USA; ^5^West Virginia University, Department of Spiritual Care and Education, One Medical Center Drive, P.O. Box 8008, Morgantown, WV 26506, USA; ^6^West Virginia University School of Medicine, Department of Medical Education, P.O. Box 9111, Robert C. Byrd Health Sciences Center, Morgantown, WV 26506, USA

## Abstract

**Background:**

Burnout and depression among physician trainees is increasing at an alarming rate. Promoting well-being is of utmost importance for graduate medical education. The primary objective was to determine if spiritual care staff/chaplaincy can assist in building emotional well-being and resiliency within medical residency education.

**Methods:**

For the academic year of July 2017 through June 2018, all graduate medical trainees in our institution were given the option of attending either an individual or group spiritual care session as part of a universal “*Call to Wellness*” curriculum. A Post-Wellness Survey was administered to measure perceptions about the program.

**Results:**

49% (*N* = 258) of residents chose to participate in a spiritual care session. Prior to the session, 51% (*N* = 132) rated their overall well-being as neutral and 25% (*N* = 64) rated their overall well-being as slightly positive, positive, or very positive. After their spiritual care session, significant improvement was seen. 25% (*N* = 64) rated their overall well-being as neutral, and 51% (*N* = 132) rated their overall well-being as slightly positive, positive, or very positive (*p* < 0.001).

**Conclusion:**

Spiritual care staff/chaplaincy can have a positive influence on emotional well-being for physicians during residency training.

## 1. Introduction

Burnout, depression, and physician suicide is a growing epidemic in the medical profession. The prevalence of burnout in resident physicians can be as high as 50% [1–3]. This is often realized early in graduate medical education (GME). Burnout and depression can have serious consequences such as reduced job performance, suboptimal patient care, and medical errors [4].

As many as 400 doctors, the equivalent of two to three graduating medical-school classes, die by suicide every year [4]. Physician workload, time pressure constraints, clerical burden, administrative tasks, and professional isolation have all been reported as factors associated with burnout [5]. Characteristics of burnout include alienation, isolation, depersonalization, cynicism toward patients, emotional exhaustion, a feeling of decreased personal achievement, a low sense of personal accomplishment at work, and a lack of empathy for patients. These characteristics can lead to a disconnect between the resident and the patient with subsequent poor patient care [4].

Hospital Spiritual Care (HSC) has been incorporated into the national care guidelines including the Joint Commission and the National Consensus Project on Quality Palliative Care [6, 7]. HSC has shown to improve the patient and family coping skills during illness and the quality of life [8, 9]. HSC offers convenient support services for patients and families and are available 24 hours a day, seven days a week.

To build resiliency in our physician trainees, the GME Wellness Taskforce in 2017 instituted a “*Call to Wellness*”. The program was intended to provide information to residents and fellows about depression and burnout, as well as to provide resources and support for those who may be suffering from these conditions. We describe our experience using hospital spiritual care staff to promote well-being amongst resident physicians training at an academic tertiary medical training center. Our objective was to determine if spiritual care staff/chaplaincy can assist in building emotional well-being and resiliency within residency education.

## 2. Materials and Methods

In 2017, physician trainees were given the option to participate in our “*Call to Wellness*” spiritual care curriculum. For those choosing to participate, they could either schedule a personal session with Hospital Spiritual Care (HSC) or attend an HSC group meeting limited to 25 physician trainees per session. Physician trainees choosing to participate were required to complete a Spiritual Care Pre-Wellness Survey exploring potential stressors followed by an hour-long individual or group session. Pre-Wellness Surveys were confidential and used for facilitation of the Spiritual Care sessions (See Supplementary [Supplementary-material supplementary-material-1]). A Post-Wellness Survey was conducted after their session to assess perceptions about the program (See Supplementary [Supplementary-material supplementary-material-1]). Local Investigative Review Board (IRB) approval was obtained.

Survey questions were based on the Patient Health Questionnaire (PHQ-9). The PHQ-9 is a validated self-administered version of the PRIME-MD diagnostic instrument to assess mental well-being. In addition to making an objective assessment of personal well-being, the PHQ-9 is also a reliable and valid measure of depression severity [10]. These characteristics plus its brevity make the PHQ-9 a useful clinical and reliable research tool.

Within the boundaries of medical ethics, HSC representatives openly encouraged religious, cultural, and/or spiritual beliefs when resident training physicians expressed a desire to include these topics in the session. Sessions also focused on resident stressors, resources for coping, and building resilience.

Descriptive statistics included frequency and valid percent for categorical items. Nominal data were analyzed using the chi-square test, with a *p* value <0.05 being statistically significant.

## 3. Results

Forty-nine percent of residents (*N* = 258) chose to participate either in an individual or group spiritual care session as part of the “*Call to Wellness*” curriculum for the academic year of July 2017 through June 2018. Prior to the session, 51% (*N* = 132) rated their overall well-being as neutral and 25% (*N* = 64) rated their overall well-being as slightly positive, positive, or very positive. After their spiritual care session, significant improvement was seen. Twenty-five percent (*N* = 64) rated their overall well-being as neutral, and 51% (*N* = 132) rated their overall well-being as slightly positive, positive, or very positive (*p* < 0.001) (Table 1).

**Figure 1:**
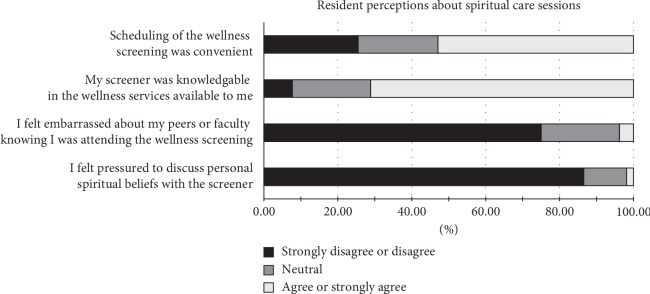
Post-Wellness Survey results.

Regarding scheduling, 53% (*N* = 137) of residents agreed or strongly agreed that scheduling was convenient. Seventy-five percent (*N* = 194) of residents said they did not feel embarrassed if their peers or colleagues knew they were attending the wellness session. Seventy-one percent (*N* = 183) felt their screener was knowledgeable about additional wellness services available to the residents. Eighty-seven percent (*N* = 224) of residents said they did not feel pressured to discuss personal spiritual beliefs with their screener, while 12% (*N* = 31) were neutral and 1% (*N* = 3) slightly agreed that they felt pressured (Figure 1).

## 4. Discussion

To our knowledge, this is the first study to assess the impact of spiritual care staff/chaplaincy as part of residency training to improve wellness. There are little data that delineate physician practices that promote successful well-being. Manusov [11] reported that religious beliefs were part of a successful strategy used by 14 first-year residents to positively influence their level of happiness. However, that study was limited to a small number of residents and only involved the first year of postgraduate training. In addition, religious beliefs were not the primary strategy but rather were included as part of other strategies such as achieving goals, being in a relationship, and receiving positive feedback. In another study, Weiner et al. [12] surveyed family medicine physicians and found that religion or spirituality correlated with improving their levels of psychological well-being. This study only involved attending physicians in a specific area of practice and not resident physicians, who are likely more susceptible to burnout and depression from a highly demanding work environment.

The participation rate of 49% (*N* = 258) may seem low; however, this goes along with other reported data. In an anonymous survey of medical doctors, Bussing et al. [13] noted that 47% are active in religious activities such as prayer, reading spiritual texts, and meditation.

We used a postintervention survey to gauge the impact of chaplaincy/spiritual care staff providing wellness interventions for training physicians. Even though wellness sessions do not have to specifically involve spiritual or religious content, hospital chaplains and/or other staff are capable of counselling resident physicians on methods to improve personal wellness.

Surveys have limitations in that respondents may not feel encouraged to take the survey, which can result in bias. In addition, respondents may not feel comfortable in providing a favorable or unfavorable response, and question interpretation may not be accurate. This may have contributed to why the remaining 51% (*N* = 132) of residents chose not to participate, although we cannot know for sure. To minimize these limitations, we used a Likert scale, which is a commonly used tool for researching popular opinion where respondents can choose an option that best supports their opinion for which they can agree or disagree with a question or statement. A control group with well-being measures provided by other wellness screeners that were not specifically hospital chaplains would have strengthened the study. Another limitation of the study is that the intervention and survey were performed at a single institution, making the results less generalizable.

## 5. Conclusion

Spiritual care staff/chaplaincy can positively influence emotional well-being for physicians during their training. Providing the resources and support needed for emotional well-being is crucial in building resiliency for resident physicians. More studies are needed to further validate this issue.

## Figures and Tables

**Table 1:** Pre- vs. Post-Wellness Survey result.

Survey response	Pre	Post
Negative	62 (24%)	62 (24%)
Neutral	132 (51%)^*∗*^	64 (25%)
Positive	64 (25%)	132 (51%)^#@^

^*∗*^ *p* < 0.01 compared to negative survey response; ^#^*p* < 0.0001 compared to group neutral survey response; ^@^*p* < 0.01 compared to negative survey response.

## Data Availability

The data used to support the findings of this study are available from the corresponding author upon request.

## Supplementary Materials

Supplementary Materials The supplementary file contains the Pre-Wellness Survey (Appendix A) and Post-Wellness Survey (Appendix B) taken by physician trainees. The “Pre” survey was used by hospital chaplains to facilitate discussion on ways to foster wellness within a physician training program. The “Post” survey assessed the effectiveness of these discussions in improving wellness.
