# Supplementary material for: Hospital Spiritual Care Can Complement Graduate Medical Trainee Well-Being
Source: Adv Med. 2019 Dec 10;2019:8749351. doi: 10.1155/2019/8749351 (PMC6925940; doi:10.1155/2019/8749351)
Supplement: Supplementary Materials — The supplementary file contains the Pre-Wellness Survey (Appendix A) and Post-Wellness Survey (Appendix B) taken by physician trainees. The “Pre” survey was used by hospital chaplains to facilitate discussion on ways to foster wellness within a physician training program. The “Post” survey assessed the effectiveness of these discussions in improving wellness. [file 8749351.f1.pdf]

Appendix A. Pre-Survey: GME Universal Wellness Screening Program

Hospital chaplains served as wellness screeners for the individual and group sessions offered to medical residents. Prior to attending an individual session, the resident completed a personal wellness assessment below that was then used by the screener to guide the session by walking through each question and facilitating conversation around how the resident assessed themselves in the various areas. The screener pointed out areas that may need attention or reflection and took the opportunity to affirm positive aspects of the assessment. The session included opportunity for the resident to share about their residency experience and each were encouraged to follow-up with wellness resources.

During a group session, the screener walked through the wellness assessment questions as a guide for group discussion, highlighting important areas for reflection and giving residents opportunities to share with one another. Several of the group sessions involved residents from the same department, which worked well in terms of facilitating connection and conversation about a shared experience amid raising one's own personal awareness of wellness. Residents were encouraged to be support for each other and given wellness resources for personal follow-up.

Wellness Survey for Medical Residents 2017-2018

Please complete the survey and send it back to the Spiritual Care Department. Your department heads will be notified that you have received the survey and they will be notified when you return the survey. All survey answers will be confidential and will be used during your half hour wellness assessment.

1. How would you describe your current mental/emotional health?

   a. Exceptional/Positive

   b. Varies/ more positive than negative

   c. Poor/ needs attention

Exploring Stressors

2. Feeling stress from a large patient load.

   a. Occasionally

   b. Frequently

   c. Most of the time

3. Feeling stress from your over all workload.

   a. Occasionally

   b. Frequently

   c. Most of the time

4. Feeling stress from moral dilemma situations in providing care.

   a. Never

   b. Occasionally

   c. Frequently

5. Feeling stressed from inadequate sleep (less than 3 hours a night).

   a. Occasionally

   b. Frequently

   c. Most of the time

6. Feeling stress from sexual harassment.

a. Never

b. Occasionally

c. Frequently

7. Feeling stress from diversity prejudices.

a. Never

b. Occasionally

c. Frequently

8. Feeling stress from caring for a difficult patient (behavioral issues).

a. Occasionally

b. Frequently

c. Most of the time

9. Feeling stressed from dealing with a difficult family.

a. Occasionally

b. Frequently

c. Most of the time

10. Feeling stress from the fear of litigation.

a. Occasionally

b. Frequently

c. Most of the time

11. Feeling stress due to fears you will become ill from a contagious illness.

    a. Never

    b. Occasionally

    c. Frequently

12. Feeling stress due to a patient death.

    a. Never

    b. Occasionally

    c. Frequently

13. Feeling stress due to lack of personal time.

    a. Rarely

    b. Occasionally

    c. Frequently

14. Feeling stress over personal/family issues.

    a. Rarely

    b. Occasionally

    c. Frequently

15. Feeling stress from inadequate sexual activity.

    a. Rarely

    b. Occasionally

    c. Frequently

16. Feeling stress from social isolation.

   a. Rarely

   b. Occasionally

   c. Frequently

17. Feeling stress from patient care situations that you have difficulty letting go of.

   a. Rarely

   b. Occasionally

   c. Frequently

Other Exploration

18. Number of meaningful adult relationships outside of work.

   a. None

   b. 1 to 4 people

   c. 5 or more

19. How would you describe the most significant relationship(s) in terms of its effect on your mood?

   a. Positive/Renewing

   b. There for me when I need them and reach out to them

   c. Not helpful

20. How would you describe the effects of your work schedule on the most significant relationship(s) in your life.

a. Minimal affect

b. Occasionally affects the relationship(s)

c. Has a detrimental effect on the relationship(s)

21. How would improvements in those significant relationship(s) benefit your emotional health? (can choose more than one)

a. I could cope with work better

b. I could sleep better

c. Would not help

22. Do you practice any form of self-care?

a. Yes

b. No

23. How would you characterize the self-care?

a. Physical activity

b. Relaxation activity

c. Escape activity (reading, gaming, etc.)

24. How effective is your current self-care?

a. Working well

b. Could use improvement

c. Not working

25. Do you have a trusted person you can talk to for dealing with stress?

a. Yes

b. No

c. Don’t need one

Appendix B. Post-Survey: GME Universal Wellness Screening Program

General

1. My current training level is:
2. This past academic year, I took advantage of the option to page the chaplain-on-call, or to schedule an appointment.
3. How would you rate your feelings about the Universal Wellness Screening Program PRIOR to your scheduled session?

   1: Very Negative 2: 3: 4: Neutral 5: 6: 7: Very Positive

Fulfilment of Screening

4. How did you fulfil your wellness screening requirement?

Explaining "Other"

5. If you chose "Other" in the previous question, please explain how you fulfilled your Universal Wellness Screening requirement?

Your personal experience

Please respond to the following statements based on your personal experience with your wellness screening:

6. I felt pressured to discuss religion or personal spiritual beliefs with my screener.

   1: Strongly Disagree 2: 3: 4: Neutral 5: 6: 7: Strongly Agree

7. I felt embarrassed about my peers or faculty knowing I was attending the wellness screening.

   1: Strongly Disagree 2: 3: 4: Neutral 5: 6: 7: Strongly Agree

8. My screener was knowledgeable in the wellness services available to me.

   1: Strongly Disagree 2: 3: 4: Neutral 5: 6: 7: Strongly Agree

9. My screener understood the stressors I face daily.

1: Strongly Disagree 2: 3: 4: Neutral 5: 6: 7: Strongly Agree

10. Scheduling of the wellness screening was convenient.

1: Strongly Disagree 2: 3: 4: Neutral 5: 6: 7: Strongly Agree

11. How would you rate your feelings about the Universal Wellness Screening Program AFTER your scheduled session?

1: Very Negative 2: 3: 4: Neutral 5: 6: 7: Very Positive

12. How likely would you be to seek help in the future through Spiritual Care or FSAP if you experienced symptoms of burnout, depression, anxiety, etc?

1: Not likely at all 2: 3: 4: Neutral 5: 6: 7: Very Likely

13. Should GME continue the Universal Wellness Screening Program?

1: Definitely Not 2: 3: 4: I'm Not Sure 5: 6: 7: Absolutely Yes

14. Please share your comments and/or suggestions regarding the Universal Wellness Screening Program.

End of Survey
